# Clinical Outcomes of Second- versus First-Generation Carotid Stents: A Systematic Review and Meta-Analysis

**DOI:** 10.3390/jcm11164819

**Published:** 2022-08-17

**Authors:** Adam Mazurek, Krzysztof Malinowski, Kenneth Rosenfield, Laura Capoccia, Francesco Speziale, Gianmarco de Donato, Carlo Setacci, Christian Wissgott, Pasqualino Sirignano, Lukasz Tekieli, Andrey Karpenko, Waclaw Kuczmik, Eugenio Stabile, David Christopher Metzger, Max Amor, Adnan H. Siddiqui, Antonio Micari, Piotr Pieniążek, Alberto Cremonesi, Joachim Schofer, Andrej Schmidt, Piotr Musialek

**Affiliations:** 1 Department of Cardiac and Vascular Diseases, John Paul II Hospital, Jagiellonian University, 31-202 Krakow, Poland; 2 Department of Bioinformatics and Telemedicine, Faculty of Medicine, Jagiellonian University Medical College, 31-008 Krakow, Poland; 3 Vascular Surgery, Surgery Department, Massachusetts General Hospital, Boston, MA 02114, USA; 4 Vascular and Endovascular Surgery Unit, Department of Surgery, Sapienza University of Rome, 00185 Rome, Italy; 5 Department of Vascular Surgery, University of Siena, 53100 Siena, Italy; 6 Institut für Diagnostische und Interventionelle Radiologie/Neuroradiologie, Imland Klinik Rendsburg, 24768 Rendsburg, Germany; 7 Department of Interventional Cardiology, John Paul II Hospital, Jagiellonian University, 31-202 Krakow, Poland; 8 Centre of Vascular and Hybrid Surgery, E.N. Meshalkin National Medical Research Center, 630055 Novosibirsk, Russia; 9 Department of General, Vascular Surgery, Angiology and Phlebology, Medical University of Silesia, 40-055 Katowice, Poland; 10 Division of Cardiology, AOR San Carlo, 20123 Potenza, Italy; 11 Wellmont CVA Heart and Vascular Institute, Kingsport, TN 37660, USA; 12 Department of Interventional Cardiology, U.C.C.I. Polyclinique d’Essey, 54270 Nancy, France; 13 Department of Neurosurgery, SUNY University at Buffalo, Buffalo, NY 14203, USA; 14 Department of Biomedical and Dental Sciences and Morphological and Functional Imaging, University of Messina, 98122 Messina, Italy; 15 Cardiovascular Department, Humanitas Gavazzeni Hospital, 24125 Bergamo, Italy; 16 MVZ-Department Structural Heart Disease, Asklepios Clinic St. Georg, 20099 Hamburg, Germany; 17 Department of Angiology, University Hospital Leipzig, 04103 Leipzig, Germany

**Keywords:** carotid artery stenting, systematic review and meta-analysis, stent design, “mesh-covered” dual-layer stents, stroke prevention

## Abstract

Background: Single-cohort studies suggest that second-generation stents (SGS; “mesh stents”) may improve carotid artery stenting (CAS) outcomes by limiting peri- and postprocedural cerebral embolism. SGS differ in the stent frame construction, mesh material, and design, as well as in mesh-to-frame position (inside/outside). Objectives: To compare clinical outcomes of SGS in relation to first-generation stents (FGSs; single-layer) in CAS. Methods: We performed a systematic review and meta-analysis of clinical studies with FGSs and SGS (PRISMA methodology, 3302 records). Endpoints were 30-day death, stroke, myocardial infarction (DSM), and 12-month ipsilateral stroke (IS) and restenosis (ISR). A random-effect model was applied. Results: Data of 68,422 patients from 112 eligible studies (68.2% men, 44.9% symptomatic) were meta-analyzed. Thirty-day DSM was 1.30% vs. 4.11% (*p* < 0.01, data for SGS vs. FGS). Among SGS, both Casper/Roadsaver and CGuard reduced 30-day DSM (by 2.78 and 3.03 absolute percent, *p* = 0.02 and *p* < 0.001), whereas the Gore stent was neutral. SGSs significantly improved outcomes compared with closed-cell FGS (30-day stroke 0.6% vs. 2.32%, *p* = 0.014; DSM 1.3% vs. 3.15%, *p* < 0.01). At 12 months, in relation to FGS, Casper/Roadsaver reduced IS (−3.25%, *p* < 0.05) but increased ISR (+3.19%, *p* = 0.04), CGuard showed a reduction in both IS and ISR (−3.13%, −3.63%; *p* = 0.01, *p* < 0.01), whereas the Gore stent was neutral. Conclusions: Pooled SGS use was associated with improved short- and long-term clinical results of CAS. Individual SGS types, however, differed significantly in their outcomes, indicating a lack of a “mesh stent” class effect. Findings from this meta-analysis may provide clinically relevant information in anticipation of large-scale randomized trials.

## 1. Introduction

Carotid artery stenting (CAS) is established as an important minimally invasive treatment modality in primary and secondary stroke prevention in atherosclerotic carotid artery disease. Meta-analyses of large-scale randomized trials of first-generation (single-layer) stent CAS versus surgery (carotid endarterectomy, CEA) demonstrated equipoise of the two treatment modalities in long-term outcomes. Nevertheless, FGS CAS has been associated with a higher rate of ipsilateral neurologic events (mainly minor strokes) than CEA [1,2]. A significant proportion of these events (≈30–60%) occurs in the postprocedural period [3,4,5,6] and has been linked to plaque prolapse through the stent struts, triggering cerebral embolism [7,8]. Although neuroprotection devices may reduce CAS embolism during the procedure [9,10,11], the brain is no longer protected against embolism after the protection device is removed [7,9,10,11,12]. After the procedure the stent plays the role of a fundamental mechanistic protector against plaque-related adverse events. Single-layer closed-cell stent design may be associated with cerebral embolism resulting from plaque prolapse [3,13,14,15].

Today, effective plaque insulation has become a leading challenge in carotid disease management using the endovascular route [15,16]. To minimize atherosclerotic plaque prolapses and reduce adverse neurologic events in CAS [16,17], mesh stents (second-generation stents, SGS) have been developed. “Mesh stents” are often considered a new “class” of carotid stents [17,18]. However, SGS show fundamental differences in (i) the stent nitinol frame construction (closed-cell in Casper/Roadsaver, open-cell in CGuard and Gore stent), (ii) mesh material (nitinol in Casper/RoadSaver, polyethylene terephthalate in MicroNet-covered CGuard stent), mesh design (braided in Casper/Roadsaver, fenestrated in Gore stent, knitted in CGuard), and (iv) the mesh position in relation to the stent frame (stent frame wrapped with mesh in the Gore and CGuard stent, the mesh placed inside the frame in Casper/RoadSaver) [19,20,21,22].

Recently, several single-cohort studies [20,23,24,25] and two randomized studies [26,27] indicated that SGS may improve CAS outcomes by limiting peri- and postprocedural embolism. However, a pilot analysis suggested that SGS may differ in their clinical outcomes [28,29]. A systematic evaluation of SGS clinical events in comparison with FGS is lacking.

We performed a systematic review and meta-analysis of clinical outcomes with SGS in relation to FGS.

## 2. Methods

**Figure 1:**
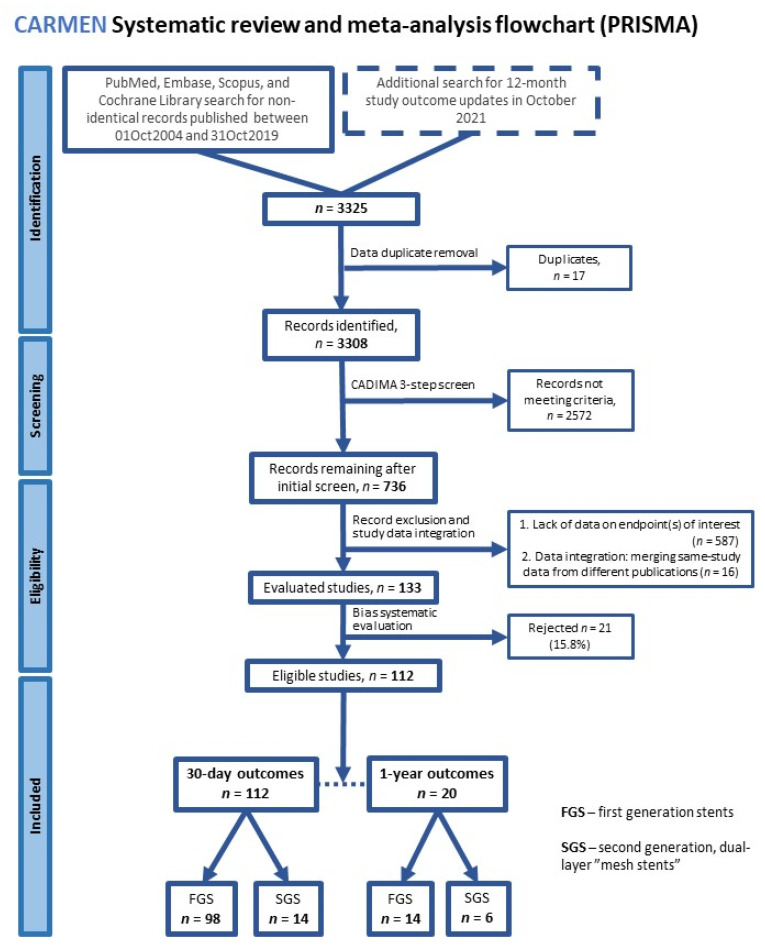
Meta-analysis flowchart. Preferred Reported Items for Systematic Reviews and Meta-Analysis (PRISMA) flowchart for studies reporting clinical outcomes of FGS and/or SGS in CAS. FGS—first-generation stent(s); SGS—second-generation stent (s); CAS—carotid artery stenting.

CAS studies with relevance to contemporary clinical practice were considered from the point of SAPPHIRE [30]. For recent studies, an 24-month period was taken from the point of 30-day data publication to capture any releases of 12-month outcomes (Figure 1).

### 2.1. Endpoints of Interest Identification

First, we assessed the clinical endpoints reported in CAS studies. A study statistician randomly identified (PubMed) 50 CAS studies reporting 30-day clinical outcomes [20,23,31,32,33,34,35,36,37,38,39,40,41,42,43,44,45,46,47,48,49,50,51,52,53,54,55,56,57,58,59,60,61,62,63,64,65,66,67,68,69,70,71,72,73,74,75,76,77,78] and 50 studies reporting 12-month clinical outcomes ([11,30,35,38,39,42,45,47,48,51,52,53,54,57,61,62,64,66,68,69,79,80,81,82,83,84,85,86,87,88,89,90,91,92,93,94,95,96,97,98,99,100,101,102,103,104,105,106,107,108]. Typically reported 30-day clinical endpoints were death (D), any stroke (S), and myocardial infarction (MI) (Figure S1A), whereas most frequently reported 1-year endpoints were ipsilateral stroke (IS) and in-stent restenosis (ISR; Figure S1B). Those endpoints were further used for data comparisons.

### 2.2. Data Search and Initial Screening

PubMed, EMBASE, and COCHRANE Library were searched for publications (1 October 2004 and 31 October 2019) using the words “carotid” + “stent” + “trial” [or] “study.” Reference lists of the identified publications were checked to capture studies not identified in the initial search, and cross-references were also used. PRISMA methodology [109] and the CADIMA tool for systematic reviews and meta-analysis [110] were applied by two independent investigators working together. Typical systematic review steps were taken, including (1) identification, (2) screening (CADIMA, full-text English language papers published in peer-reviewed journals; Figure S2), (3) eligibility check, and (4) quality assessment (Figure 1). The study was registered with the PROSPERO database of systematic reviews (CRD42022339789).

### 2.3. Study Eligibility and Quality Assessment

Studies eligible for screening needed to satisfy the criteria of at least 30 subjects, de novo atherosclerosis, extracranial carotid procedure, elective carotid procedure, and unselected population (Figure S2). Both prospective (observational and randomized) and retrospective studies were considered.

A total of 3325 records were initially identified. Of these, 3308 records (17 duplicates eliminated) were introduced to CADIMA. Besides the initial screening criteria, the following requirements were applied: (1) publication in English, (2) original study publication, (3) human subjects, (4) stenosis ≤99%, (5) transfemoral access, and (6) not a substudy of a previously published study; this led to 736 records. Data flow through the CADIMA tool is presented in Figure S2. Studies reporting the endpoints of interest (*n* = 149) were taken for further analysis. Quality assessment was performed to identify bias in at least one of five bias categories (patient selection/recruitment, performance in relation to study device(s), performance other than in relation to study device(s), outcome detection, and attrition and reporting; Figure S3). Severe bias presence led to study exclusion from further analysis. Finally, CAS data from103 observational and 9 randomized studies were included [4,21,22,23,26,27,30,35,39,41,44,45,56,58,65,66,84,89,91,92,97,104,111,112,113,114,115,116,117,118,119,120,121,122,123,124,125,126,127,128,129,130,131,132,133,134,135,136,137,138,139,140,141,142,143,144,145,146,147,148,149,150,151,152,153,154,155,156,157,158,159,160,161,162,163,164,165,166,167,168,169,170,171,172,173,174,175,176,177,178,179,180,181,182,183,184,185,186,187,188,189,190,191,192,193,194,195,196,197,198,199,200,201,202,203,204,205,206,207,208,209,210,211,212,213,214,215,216,217,218,219,220,221,222,223,224].

### 2.4. Data Extraction

Data were extracted by two investigators working together using a predefined data extraction form. In case of disagreement, a third investigator reviewed the publication(s) pertaining to a given study, and a consensus was reached.

### 2.5. Data Synthesis

The baseline demographics and outcomes were extracted. In the case of more than one publication referring to 30-day or 12-month outcomes from a particular study, the data were integrated. 

### 2.6. Statistical Analysis

Clinical characteristics of patients enrolled in meta-analyzed studies are provided as counts and percentages and (weighted) proportions for nominal variables and means with standard deviations for continuous variables. Endpoints of interest are presented as counts as well as risk ratios (95%CI) for between-group comparisons. Raw, untransformed proportions were analyzed using the DerSimonian–Laird random-effect model. The influence of covariates was assessed using metaregression. Meta-analysis results were presented as forest plots with RR (CI) for SGS (and components) compared with FGS. Publication bias was assessed using funnel plots accompanied by Egger’s regression test for asymmetry. Heterogeneity among meta-analyzed studies was presented as a fraction of variance due to heterogeneity (I^2^) and an estimate of the between-study variance (τ^2^) with *p*-value of a Q test. The continuity correction for zero-event arms was applied were applicable. Statistical analyses were performed using R v.4.1.1 (The R Foundation for Statistical Computing; https://www.r-project.org) with the “meta” package v.5.1.

## 3. Results

### 3.1. Eligible Trials and Results Display

Three-step screening followed by eligibility assessment of each record revealed 112 studies [4,21,22,23,26,27,30,35,39,41,44,45,56,58,65,66,84,89,91,92,97,104,111,112,113,114,115,116,117,118,119,120,121,122,123,124,125,126,127,128,129,130,131,132,133,134,135,136,137,138,139,140,141,142,143,144,145,146,147,148,149,150,151,152,153,154,155,156,157,158,159,160,161,162,163,164,165,166,167,168,169,170,171,172,173,174,175,176,177,178,179,180,181,182,183,184,185,186,187,188,189,190,191,192,193,194,195,196,197,198,199,200,201,202,203,204,205,206,207,208,209,210,211,212,213,214,215,216,217,218,219,220,221,222,223,224] with a total of 68,422 patients (68.2% men, 44.9% symptomatic) (Figure 1). Clinical characteristics of the patient groups in respective stent categories (FGS, SGS, and FGS separated into open-cell and closed-cell single-layer stents) are presented in Table 1. 

**Figure 2:**
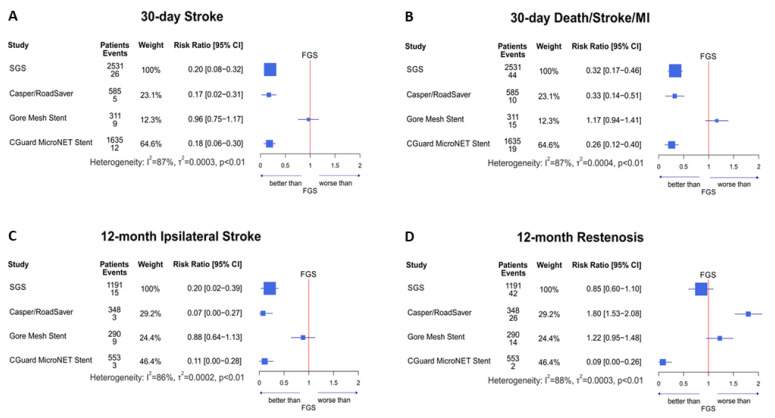
Forrest-plots for 30-day and 12-month fundamental outcomes of a meta-analysis comparing dual-layer, “mesh stents” (second-generation) vs single-layer (first-generation) carotid stents in stroke prevention. Forest plots show the data of 68,043 patients (44,9% symptomatic) included in 112 eligible studies meta-analyzed using a random-effect model. First-generation (single-layer; FGS) stent outcomes were used as a reference for the second-generation (SGS; “mesh stent”) stent effect (risk ratio, 95% CI). Clinical endpoints of interest were 30-day stroke (**A**), 30-day death/stroke/MI (**B**), 12-month ipsilateral stroke (**C**), and 12-month in-stent restenosis (**D**). Data are given for pooled SGS outcomes (top rows in A–D), followed by outcomes for individual SGS types (Casper/Roadsaver, Gore Mesh Stent, and CGuard MicroNet Stent). SGS pooled use was associated with improved short- and long-term clinical results of CAS. Individual SGS types, however, differed in their outcomes. Casper/Roadsaver and CGuard MicroNet stents were similarly effective in 30-day stroke (**A**) and death/stroke/MI reduction (**B**), whereas the Gore stent was neutral. The stent type effect on the 12-month ipsilateral stroke relative risk was consistent with the 30-day data (**C**). In contrast, the 12-month restenosis rate in relation to FGS was reduced with the CGuard MicroNet stent but increased by Casper/Roadsaver (**D**). These findings indicate a lack of “mesh stent” class effect. Absence of any SGS ‘class effect’ may result from the fundamental differences in SGS stent designs; for funnel plots, see Figures S5 and S6. Within the limitations inherent in any meta-analytic approach, these findings may inform clinical decision-making in anticipation of further head-to-head large-scale randomized trials powered for clinical endpoints. See the text for details. FGS, first-generation stent(s); SGS, second-generation stent(s).

**Figure 3:**
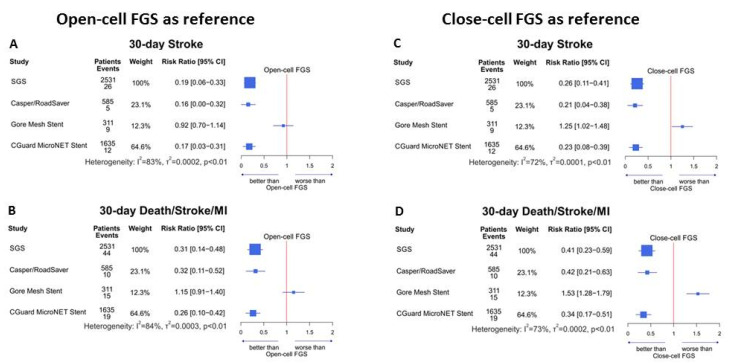
SGS 30-day clinical outcomes in relation to open-cell FGS (**A**,**B**) and closed-cell FGS (**C**,**D**) used as a reference. The forest plots include the data of 28,274 patients in studies with clinical outcomes available according to FGS stent design (i.e., open- or closed-cell FGS, left and right panel, respectively) that are used here as a reference for the SGS relative benefit/harm. SGS as a group (*n* = 2531) showed a benefit in 30-day stroke and 30-day death/stroke/MI relative risk reduction in relation to not only open- (**A**,**B**) but also closed-cell FGS (**C**,**D**). Note that this effect was driven by the Casper/Roadsaver and CGuard MicroNet stents, whereas the Gore Mesh stent was neutral in relation to open-cell FGS but came out inferior in comparison to closed-cell (see text for details). For respective funnel plots, see Supplementary Figure S5. FGS, first-generation stent(s); SGS, second-generation stent(s).

Clinical event rates (i.e., combined and individual stent-type DSM at 30 days and combined and individual 12-month IS/ISR) according to the meta-analytic model are given in Table 2. Data are given for (i) FGS vs. pooled SGS and (ii) FGS vs. each individual SGS, i.e., Casper/Roadsaver (CR), Gore stent (GS), and CGuard MicroNet-covered stent (CG). Table 3 provides a comparison of the *p*-values. The 30-day and 12-month relative outcomes for fundamental comparisons are provided in the Figure 2. Figure 3 shows the 30-day SGS outcome comparisons against the open- and closed-cell single-layer stents. The combined 12-month IS/ISR for SGS vs. FGS is presented in the Supplementary Materials (Figure S4). Funnel plots are provided in Figures S5 and S6.

### 3.2. Quality Assessment and Risk of Bias

A severe bias in at least one category, leading to study exclusion, was identified in 21 out of 133 studies (15.9%; Figure S3A). Severe bias occurred in the following categories (in order of prevalence): (i) patient selection/recruitment, (ii) outcome detection, (iii) performance unrelated to the study device, (iv) performance in relation to the study device(s), and (v) attrition and reporting (respectively 57.1%, 28.6%, 14.3%, 9.6%, and 9.6% of rejected studies; Figure S3B). Severe bias in two or more categories occurred in four (19%) rejected studies.

The overall quality of 112 included studies was moderate. Moderate bias in at least one category was present in 102 (91%) studies and in two or more categories in 64 (57%) studies (Figure S3C–E). There were 10 studies (9%) with mild or absent bias in all categories.

### 3.3. 30-Day Outcomes: SGS vs. FGS

According to the meta-analytic model, the 30-day death, stroke, and MI rate (DSM) for FGS was 4.11% (Table 2). The 30-day FGS stroke rate was 3.01% (Table 2). Pooled SGS showed a markedly lower 30-day event rate (DSM 1.30%, stroke 0.6%, absolute reduction by 2.81% and 2.41%, respectively, *p* < 0.001 vs. FGS for both; RRs and 95% CIs are given in forest plots). Individual SGS 30-day event rates were the following: CR-DSM 1.33% (*p* = 0.02 vs. FGS, absolute reduction by 2.78%), CG-DSM 1.08% (*p* < 0.001 vs. FGS, absolute reduction by 3.03%), GS-DSM 4.82% (*p* = 0.75 vs. FGS, absolute increase by 0.71%). The 30-day stroke rate was 0.5% with CR (*p* = 0.01 vs. FGS, absolute reduction by 2.51%), 2.89% with GS (*p* = 0.95 vs. FGS, absolute reduction by 0.12%), and 0.54% with CG (*p* = 0.002 vs. FGS, absolute reduction by 2.47%). The Figure 2 forest plots A and B demonstrate the 30-day relative outcomes for SGS as a group vs. FGS as well as individual SGS (CR, CG, GS) outcomes in relation to FGS.

### 3.4. 12-Month Outcomes: SGS vs. FGS

The 12-month IS rate for FGS was 3.51%. The 12-month ISR rate for FGS was 3.97% (Table 2). Pooled SGSs showed a markedly lower 12-month IS rate (0.7%, absolute reduction by 2.81%, *p* = 0.001) but not ISR reduction (3.38%, absolute reduction by 0.59%, *p* = 0.57).

Individual 12-month SGS event rate analysis revealed significant differences between the SGS types. CR-IS is 0.26% (*p* = 0.007 vs. FGS, absolute reduction by 3.25%) and CG-IS 0.38% (*p* = 0.013 vs. FGS, absolute reduction by 3.13%), GS-IS 3.1% (*p* = 0.846 vs. FGS, absolute reduction by 0.41%). The individual 12-month SGS ISR rate was 7.16% with CR (*p* = 0.04 vs. FGS, absolute increase by 3.19%), 4.83% with GS (*p* = 0.66 vs. FGS, absolute increase by 0.86%), and 0.34% with CG (*p* = 0.009 vs. FGS, absolute reduction by 3.63%). Figure 2 forest plots C and D show the 12-month relative outcomes for SGS taken as a group and for individual SGS stent brands in relation to FGS.

The 12-month combined endpoint of IS and ISR (Table 2; Figure S4) was reduced with SGSs taken as a group by 3.03% (FGS 8.15%, SGS 5.12%, *p* = 0.027). Individual SGS evaluation showed a significant reduction in IS/ISR only with CG (combined event rate 0.73%, reduction by 7.42% vs. FGS, *p* = 0.001). In contrast, CR and GS did not reduce 12-month IS/ISR against FGS (−0.29% and −0.22%; *p* = 0.99 and *p* = 0.96, respectively). In CR, the lack of a significant reduction in the 12-month combined endpoint was driven by an increase in ISR that offset the relative benefit in IS (Table 2; Figure 2 and Figure S4). For GS, the increase in combined IS/ISR (Figure S4) occurred as a result of an increase in both composites of the combined 12-month endpoint (Table 2).

### 3.5. FGS Stent Type: Open- vs. Closed-Cell Design

SGS 30-day outcome comparisons against open- and closed-cell FGS are shown in Figure 3. According to the meta-analytic model, the 30-day DSM for open-cell FGS was 4.18%, whereas for closed-cell FGS, it was 3.15%; these were reduced with SGS by 2.88% (*p* < 0.001) and 1.85% (*p* = 0.005), respectively. The 30-day stroke rate was 3.15% for open-cell FGS and 2.32% for closed-cell FGS (reduction with SGS respectively by 2.55%, *p* < 0.001; and 1.72%, *p* = 0.005).Thirty-day DSM comparisons for individual SGS brands against open-cell FGS showed the following: an absolute reduction of 2.85% for CR (*p* = 0.004), a nonsignificant absolute increase by 0.64% for GS (*p* = 0.73), and an absolute 3.1% for CG (*p* < 0.001). Thirty-day DSM comparisons for individual SGS brands against closed-cell FGSs showed an absolute reduction by 1.82% for CR (*p* = 0.030), an absolute increase by 1.67% for GS (*p* = 0.031), and an absolute reduction by 2.07% for CG (*p* = 0.003).

Thirty-day stroke rate comparisons for individual SGS against closed-cell FGS showed an absolute reduction of 2.65% for CR (*p* = 0.001), a nonsignificant reduction by an absolute 0.26% for GS (*p* = 0.88), and an absolute reduction by 2.61% for CG (*p* < 0.001). Thirty-day stroke rate comparisons for individual SGS against closed-cell FGS showed a reduction by 1.82% for CR (*p* = 0.02), an increase by 0.57% for GS (*p* = 0.036), and a reduction by 1.78% for CG (*p* = 0.01).

Overall, SGS individual and group 30-day outcomes were consistent irrespective of open- or closed-cell FGS use as a comparator. There were not enough studies reporting 12-month outcomes of open- and closed-cell FGS to enable a separate 12-month clinical endpoint evaluation of SGS in relation to open- and closed-cell FGS.

### 3.6. SGS Stent Brand Comparisons

The comparison of 30-day and 12-month outcomes within the SGS group revealed marked differences between the individual SGS representatives (Table 2). The 30-day DSM and stroke rate were similar between CR and CG (1.33% and 1.08%, nonsignificant increase in CG of 0.25%, *p* = 0.37). GS, however, showed an increase in 30-day DSM (4.82%) compared with both CR (significant increase of 2.39%, *p* = 0.001) and CG (significant increase of 2.35%, *p* = 0.01). The 30-day stroke rate was not different between CR and CG (0.50% and 0.54%, respectively, nonsignificant increase in CG of 0.04%, *p* = 0.899), but was significantly higher in GS compared with both CR and CG (2.89%; an increase by an absolute 2.39% and 2.35%, *p*= 0.017 and *p* = 0.045, respectively).

The 12-month IS/ISR rate in CG (0.73%) was significantly lower than in CR or GS (7.86% and 7.93%; reduction by an absolute 7.13% vs. CR and by 7.20% vs. GS; *p* < 0.001 and *p* = 0.01, respectively). There was no difference in the 12-month IS/ISR between CR and GS (nonsignificant reduction by 0.07% for CR, *p* = 0.80). This was driven by a significantly lower 12-month ISR in CG (0.34%) in relation to CR (7.16%, reduction of 6.82%, *p* < 0.001) and GS (4.83%, reduction of 4.49%, *p* = 0.01) and no difference between CR and GS (ISR reduction with GS against CR by 2.33%, which did not reach statistical significance, *p* = 0.34). The 12-month IS rate was similar for CR and CG (0.26% and 0.38%, *p* = 0.717) but was higher in GS (3.1%, a significant increase by an absolute 2.84% vs. CR, *p* = 0.014; and an increase by an absolute 2.72% with GS vs. CG; Table 2).

### 3.7. Heterogeneity

There was considerable heterogeneity among the analyzed studies and the outcomes of interest (I^2^ > 70% for all outcomes), prompting the use of a random-effect meta-analytic model. Heterogeneity evaluation using the Q test was concordant (*p* < 0.01 for all analyzed studies/outcomes). Funnel plots are provided in Figure S5.

## 4. Discussion

The fundamental findings from this systematic review and meta-analysis comparing second-generation (“mesh stent”) against first-generation (single metallic layer stent) are the following: (1) The 30-day death/stroke/MI rate were significantly reduced with SGS, an effect driven predominantly by a reduction in peri- and postprocedural strokes with CGuard and Casper/Roadsaver. (2) Among the SGSs, both Casper/Roadsaver and CGuard reduced the 30-day DSM and stroke rates, whereas the Gore stent was neutral. (3) SGS showed superiority also when compared with closed-cell FGS, including a nearly four-fold reduction in 30-day strokes. (4) At 12 months, in relation to FGS, Casper/Roadsaver reduced IS but increased ISR, CGuard showed a reduction in both IS and ISR, and the Gore stent was neutral.

The stent in carotid artery intervention plays a unique role in that after the embolic protection system has been removed, the stent is the main line of defense (along with antiplatelet therapy) against embolic and thromboembolic complications that may arise from the newly remodeled plaque with the varying degree of plaque coverage dependent on the stent design [225].

This work was undertaken to generate information with respect to patient outcomes that are relevant in routine clinical practice. Today, clinicians are exposed to often contradictory data regarding strategies in carotid revascularization in primary and secondary stroke prevention. Within the limitations that need to be taken into account (see below), the data from this systematic review and meta-analysis may play a role in informing clinical decisions until larger sets of randomized evidence [27] become available.

There was a considerable heterogeneity among the analyzed studies and the outcomes of interest (I^2^ > 70%, *p* < 0.01 in Q test). Although a lower level of heterogeneity would be considered optimal for overall data interpretation, what this work found is a reflection of reality as per a rigorous process of data identification and quality assessment. Several factors may contribute to high heterogeneity within the pool of CAS data available today. These include differences in study populations, different specialties performing the procedures (resulting in differences in patient selectionfor CAS), differences in study design (such as randomized or single cohort), some changes in clinical guidelines and definitions over time, and evolution in pharmacotherapy and medical equipment used in CAS. All those may be relevant to this analysis even if we have not taken into consideration data from before the SAPPHIRE study [30] that may have less relevance to contemporary clinical practice. 

Overall, the heterogeneity level of studies in this systematic review and meta-analysis is considered to reflect the large spectrum of patients treated with CAS, with variations in the proportions of symptomatic and asymptomatic patients.

Indexes of data heterogeneity in this meta-analysis prompt caution in interpreting the results. Nonetheless, when considering the relevance of this work to clinical decision-making, it is important to note that the event rates in upcoming studies of FGS and SGS are broadly concordant with the event rates indicated in this meat-analysis (Table 2). Contemporary FGS data in the ACST-2 trial CAS arm (1811 patients with asymptomatic carotid stenosis, >98% FGS use) show the 30-day DSM of 3.9% (30-day stroke rate, 3.6%) [226].

Most recent an upcoming studies show event rates consistent with the meta-analytic model. Regarding SGS, the most recent Casper/Roadsaver and CGuard data show 30-day and 12-month event rates consistent with those indicated by the meta-analytic model. Some exception is the 30-day DSM with Casper/Roadsaver; that in some reports, appears to be higher than indicated on the basis of initial data sets. Specifically, in a recent study of 287 patients implanted with Casper/Roadsaver, there were nine strokes by 30 days (3.1%), including three postprocedural ischemic strokes (two due to stent thrombosis) [227], a rate greater than that indicated by our random-effects model (Table 2). Regarding 12-month outcomes with Casper/Roadsaver, recent multicentric data from Japan show an ISR rate of 8.5% and a 12-month IS/ISR rate of 9.9% [228], consistent with the rate determined by this meta-analysis (7.86, 95%CI 5.04–10.68; Table 2). Another very recent study reported a Casper/Roadsaver ISR rate of 8.2% at 12 months that further increased to 13.3% at 2 years [229].

Upcoming data regarding the real-life performance of the CGuard stent are consistent with the findings from this meta-analysis. In 103 patients recently treated with CGuard, no DSM occurred by 30 days [230]. A very recent 733-patient multicentric (20 centers) CGuard study in Italy showed three strokes by 30 days (0.4%, cumulative DSM rate of 0.95%) [231] and a 12-month ISR rate of 0.82% [232]. These outcomes are consistent with those indicated by the meta-analytic model (Table 2). As the Gore mesh stent has not been marketed, data other than captured in this systematic review are not available.

What is needed next is (i) to rigorously compare SGS outcomes against contemporary surgery and the hybrid carotid revascularization technique of transcarotid revascularization (TCAR) using a conventional (single-layer) carotid stent and (ii) to evaluate long-term outcomes with SGSs [178]. SGS comparisons against contemporary carotid endarterectomy that shows a 30-day DSM of ≈1.9% [233] is particularly needed. Very relevant in the context of the present analysis are the TCAR data, demonstrating that despite optimized intraprocedural cerebral protection, the use of FGS in TCAR is associated with a two-fold increase in early stroke/TIA in symptomatic vs. asymptomatic patients (2.5% vs. 1.2%, odds ratio 1.99, 95% CI 1.01–3.92, *p* = 0.046) [234]. This suggests that SGS plaque sealing might improve TCAR outcomes in symptomatic patients and high-risk lesions in particular [235]. Rigorous follow-up of SGS-implanted patients beyond 12 months is also needed [178,229,236].

## 5. Limitations

One fundamental limitation of this meta-analysis is a large disproportion between the volume of SGS vs. FGS data. This, however, is natural with any new technology that requires to be compared with a historical standard. Secondly, the majority of SGS studies were performed later than FGS studies; thus, the evolution of pharmacologic and interventional techniques (and experience of operators) might affect the outcomes. Third (and for the reasons above), this work is based mostly on single-arm studies and stent arm data from trials comparing CAS with surgery. Regrettably, no sufficient patient characteristics information was routinely provided to enable propensity matching. Fourth, relative differences in the volume of individual stent type (or brand) data published within the particular group(s) may contribute to the “direction” of the overall group data reflecting the largest component of the group. This may be relevant particularly for the SGS pooled results where individual stent type outcomes differ. While this cannot be corrected by matching the group volumes (as a systematic review of published data, by definition needs to include all studies that meet the search criteria), it is elucidated by providing individual SGS stent type comparisons – both against FGS (Figure 1 and Figure 2) and among the SGS group (Table 2). Fifth, with >50,000 patients analyzed (112-studies), it was not feasible to obtain and process individual patient data [28,29]. Similarly, it was not possible to analyze the technical success rate (or procedural difficulties with any particular stent types), particularly as these (unfortunately) do not get routinely reported. Stent design-related differences, including the delivery profile and properties of the individual stent delivery systems, may play a practical role particularly for some less experienced operators. Sixth, there have been several changes in MI definition over time, possibly affecting the DSM endpoint in our analysis; this, however, would favor FGS rather than SGS. Seventh, consistent with prior analyses [235,237], there were not enough studies to analyze SGS 12-month outcomes against 12-month outcomes separately for open- and closed-cell single-layer stents. Eighth, although studies with a clear bias were excluded (21/133, 15.8%), the overall quality of the published data was found to be moderate. Finally, the findings from the present analysis may be affected by selective reporting and publication bias.

## 6. Conclusions

A systematic review and meta-analysis of available data indicates that SGS use may be associated with significantly better (than FGS) short- and long-term results of CAS, providing meta-analytic evidence for improvement in CAS outcomes with dual-layer stent technologies [238]. The SGS benefit is particularly relevant were both 30-day and 12-month rate of complications is reduced (Figure 2). An important finding is that the individual SGS types significantly differ (both in their outcomes related to FGS and for outocmes within the SGS group) indicating lack of any carotid ‘mesh-stent’ class effect. This work provides several clinically-relevant hypotheses for further testing in large randomized trials powered for clinical endpoints. However, in absence of large-scale randomized evidence at present, data from this systematic review and meta-analysis may inform clinical decision-making regarding device choices in percutaneous carotid revascularization.

## 7. Perspectives

### 7.1. What Is Known?

Several single-cohort studies have suggested that second-generation stents (SGS; “mesh stents”) may improve carotid artery stenting (CAS) outcomes by limiting peri- and inhibiting postprocedural cerebral embolism. A recent randomized controlled study demonstrated a profound reduction in periprocedural (and elimination of postprocedural) cerebral embolism with the MicroNet-covered stent in relation to a first-generation (FGS; single metallic layer) stent [27].

“Mesh stents” differ in the stent frame construction, mesh material, and design, as well as mesh-to-frame placement (mesh wrapping the stent frame vs. placed inside).

### 7.2. What Is New?

Our systematic review and meta-analysis of the clinical data of 68,422 patients (112 studies) treated using FGS or SGS demonstrated that outcomes at 30 days (death/stroke/MI) were significantly improved for pooled “mesh stents” in relation to FGSs. The benefit was present for SGSs against both open and closed-cell FGS. At 12 months, ipsilateral stroke and in-stent restenosis were significantly reduced with SGS. However, individual SGS significantly varied in their performance at 30-days and 12-months, indicating a lack of a “class” effect. This may be relevant for decision-making in primary and secondary stroke prevention with CAS in clinical practice.

### 7.3. What Is Next?

While upcoming studies of FGS and SGS show outcomes largely consistent with this meta-analysis, large-scale randomized controlled studies powered for clinical outcomes would be ideally desired for a rigorous prospective comparison of individual SGS types against FGS and against surgery.

## Figures and Tables

**Table 1:** Clinical characteristics of meta-analyzed groups.

	FGS	SGS	*p* FGS vs. SGS	Open-Cell FGS	Closed-Cell FGS	*p* Open-Cell vs. Closed-Cell FGS	*p* Open-Cell FGS vs. SGS	*p* Closed-Cell FGS vs. SGS
**No. of studies**	98	14	-	29	12	-	-	-
**No. of patients**	65,891	2531	-	21,351	7598	-	-	-
**Age (SD)**	70.1 (2.8)	71.9 (2.5)	0.02	70.4 (3.2)	69.3 (3.4)	0.60	0.32	0.13
**Male**	68%	73%	0.046	68%	66%	0.92	0.12	0.15
**Symptomatic**	45%	41%	0.40	43%	50%	0.61	0.94	0.45
**Diabetic**	34%	32%	0.43	35%	36%	0.71	0.88	0.61
**CAD**	51%	47%	0.55	48%	55%	0.59	0.98	0.98
**AF**	6%	3%	0.37	3%	ND	-	0.99	-
**Contralateral occlusion**	10%	16%	0.22	10%	12%	0.87	0.63	0.99
**Embolic protection in CAS**	95.8%	97.1%	0.656	97.3%	99.4%	0.09	0.85	0.2

Data are shown as absolute number, mean (SD), or weighted proportion (%) as appropriate.

**Table 2:** The 30-day and 12-month event rates by stent type (random-effect model).

	FGS	SGS	Casper/ Roadsaver	Gore	CGuard
**30-day** **Stroke** (%) (95% CI)	**3.01** (2.63–3.38)	**0.60** (0.28–0.92)	**0.50** (0–1.15)	**2.89** (1.03–4.76)	**0.54** (0.17–0.92)
**30-day** **Death/Stroke/MI** (%) (95% CI)	**4.11** (3.65–4.56)	**1.30** (0.64–1.96)	**1.33** (0–2.66)	**4.82** (2.44–7.2)	**1.08** (0.55–1.60)
**12-mo** **Ipsilateral Stroke** (%) (95% CI)	**3.51** (2.52–4.50)	**0.7** (0–1.47)	**0.26** (0–1.27)	**3.1** (1.11–5.1)	**0.38** (0–0.9)
**12-mo** **Restenosis** (%) (95% CI)	**3.97** (0.28–5.14)	**3.38** (1.39–5.37)	**7.16** (5.45–9.86)	**4.83** (2.36–7.29)	**0.34** (0–0.82)
**12-mo** **Ipsilateral Stroke/Restenosis** (%) (95% CI)	**8.15** (6.63–9.96)	**5.12** (3.14–6.10)	**7.86** (5.04–10.68)	**7.93** (4.82–11.04)	**0.73** (0–1.44)

**Table 3:** The *p*-values for 30-day and 12-mo SGS event rate comparisons against FGS (for the meta-analytic model raw event rates, see Table 2).

	*p* FGS vs. SGS	*p* *FGS* vs. Roadsaver	*p* FGS vs. Gore	*p* FGS vs. CGuard
**30-day** **Stroke**	**<0.001**	**0.011**	0.954	**0.002**
**30-day** **Death/Stroke/MI**	**<0.001**	**0.022**	0.750	**<0.001**
**12-mo Ipsilateral Stroke**	**0.001**	**0.007**	0.846	**0.013**
**12-mo Restenosis**	0.569	**0.041**	0.658	**0.009**
**12-mo Ipsilateral Stroke/Restenosis**	0.027	0.998	0.961	**0.001**

## Data Availability

Raw extracted data are available (on request) from the corresponding authors (A.M. and P.M.); statistical analysis details are available (on request) from the study statistician (K.M.).

## Supplementary Materials

The following supporting information can be downloaded at: https://www.mdpi.com/article/10.3390/jcm11164819/s1, Figure S1. Random Sample CAS Study Endpoints. (A) for 30-day endpoints (*n* = 50) and (B) for 12-month endpoints (*n* = 50); Figure S2. Study selection process: CADIMA systematic review and mata-analysis tool; Figure S3. Bias systematic assessment; Figure S4. Forrest-plot presenting meta-analytic data for the combined 12-month endpoint of ipsilateral stroke and restenosis. With FGS used as a reference, the benefit of Casper/Roadsaver in reducing 12-month ipsilateral stroke rate was neutralized by its relative harm—increased restenosis rate (for the individual endpoint data see Figure 2); Figure S5. Funnel plots of different stent type comparisons—30-day outcomes; Figure S6. Funnel plots of different stent type comparisons-12-monthy outcomes.

## Abbreviations

CADIMA	Online evidence synthesis tool for the conduct and reporting of systematic reviews
CG	CGuard MicroNet-covered carotid stent (laser-cut nitinol frame covered with PET micro-mesh sleeve)
DSM	Death, stroke, myocardial infarction
FGS	First-generation (single-layer) carotid stent(s)
GS	Gore carotid stent (laser-cut nitinol frame covered by Teflon mesh layer)
IS	Ipsilateral stroke
ISR	In-stent restenosis
CR	Casper/RoadSaver dual metallic layer carotid stent (braided metallic mesh *inside* the metallic, braided frame)
PRISMA	Preferred Reporting Items for Systematic Review and Meta-Analysis
PROSPERO	International Prospective Register of Systematic Reviews
SGS	Second-generation (mesh-stent) carotid stent(s)
