# Supplementary material for: Clinical Outcomes of Second- versus First-Generation Carotid Stents: A Systematic Review and Meta-Analysis
Source: J Clin Med. 2022 Aug 17;11(16):4819. doi: 10.3390/jcm11164819 (PMC9409706; doi:10.3390/jcm11164819)
Supplement: Supplementary file 1 [file jcm-11-04819-s001.zip › jcm-1800685-supplementary.pdf]

Journal of
Clinical Medicine

SUPPLEMENTARY MATERIAL:

Systematic Review

Clinical Outcomes of Second- versus First-Generation Carotid Stents: A Systematic Review and Meta-Analysis

Adam Mazurek [1,*], Krzysztof Malinowski [2], Kenneth Rosenfield [3], Laura Capoccia [4], Francesco Speziale [4], Gianmarco de Donato [5], Carlo Setacci [5], Christian Wissgott [6], Pasqualino Sirignano [4], Lukasz Tekieli [7], Andrey Karpenko [8], Waclaw Kuczmik [9], Eugenio Stabile [10], David Christopher Metzger [11], Max Amor [12], Adnan H. Siddiqui [13], Antonio Micari [14], Piotr Pieniążek [1,7], Alberto Cremonesi [15], Joachim Schofer [16], Andrej Schmidt [17] and Piotr Musialek [1,*] on behalf of CARMEN (CArotid Revascularization systematic reviews and MEta-aNalyses) Investigators [†]

1 Department of Cardiac and Vascular Diseases, John Paul II Hospital, Jagiellonian University, 31-202 Krakow, Poland
2 Department of Bioinformatics and Telemedicine, Faculty of Medicine, Jagiellonian University Medical College, 31-008 Krakow, Poland
3 Vascular Surgery, Surgery Department, Massachusetts General Hospital, Boston, MA 02114, USA
4 Vascular and Endovascular Surgery Unit, Department of Surgery, Sapienza University of Rome, 00185 Rome, Italy
5 Department of Vascular Surgery, University of Siena, 53100 Siena, Italy
6 Institut für Diagnostische und Interventionelle Radiologie/Neuroradiologie, Imland Klinik Rendsburg, 24768 Rendsburg, Germany
7 Department of Interventional Cardiology, John Paul II Hospital, Jagiellonian University, 31-202 Krakow, Poland
8 Centre of Vascular and Hybrid Surgery, E.N. Meshalkin National Medical Research Center, 630055 Novosibirsk, Russia
9 Department of General, Vascular Surgery, Angiology and Phlebology, Medical University of Silesia, 40-055 Katowice, Poland
10 Division of Cardiology, AOR San Carlo, 20123 Potenza, Italy
11 Wellmont CVA Heart and Vascular Institute, Kingsport, TN 37660, USA
12 Department of Interventional Cardiology, U.C.C.I. Polyclinique d'Essey, 54270 Nancy, France
13 Department of Neurosurgery, SUNY University at Buffalo, Buffalo, NY14203, USA
14 Department of Biomedical and Dental Sciences and Morphological and Functional Imaging, University of Messina, 98122 Messina, Italy
15 Cardiovascular Department, Humanitas Gavazzeni Hospital, 24125 Bergamo, Italy
16 MVZ-Department Structural Heart Disease, Asklepios Clinic St. Georg, 20099 Hamburg, Germany
17 Department of Angiology, University Hospital Leipzig, 04103 Leipzig, Germany
* Correspondence: mazurekadam@yahoo.pl (A.M.); pmusialek@szpitaljp2.krakow.pl (P.M.)
† CARMEN Investigators Membership are provided in the Supplementary Material.

Citation: Mazurek, A.; Malinowski, K.; Rosenfield, K.; Capoccia, L.; Speziale, F.; de Donato, G.; Setacci, C.; Wissgott, C.; Sirignano, P.; Tekieli, L.; et al. Clinical Outcomes of Second- versus First-Generation Carotid Stents: A Systematic Review and Meta-Analysis. *J. Clin. Med.* **2022**, *11*, x. https://doi.org/10.3390/xxxxx

Academic Editors: George N. Kouvelos

Received: 18 June 2022
Accepted: 29 July 2022
Published: date

Publisher's Note: MDPI stays neutral with regard to jurisdictional claims in published maps and institutional affiliations.

Copyright: © 2022 by the authors. Submitted for possible open access publication under the terms and conditions of the Creative Commons Attribution (CC BY) license (https://creativecommons.org/licenses/by/4.0/).

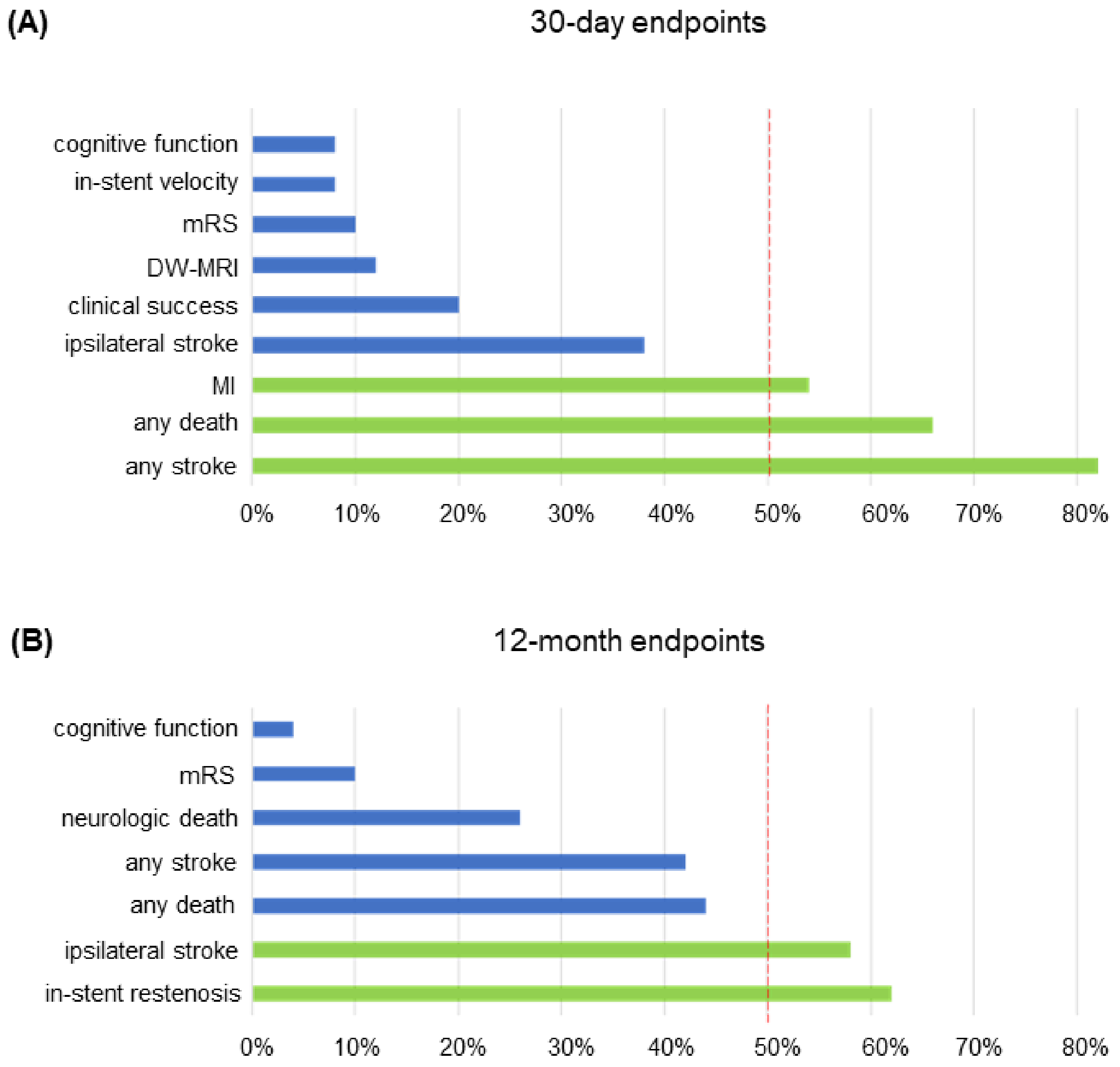

Figure S1. Random Sample CAS Study Endpoints. (A) for 30-day endpoints (n=50), and (B) for 12-month endpoints (n=50).

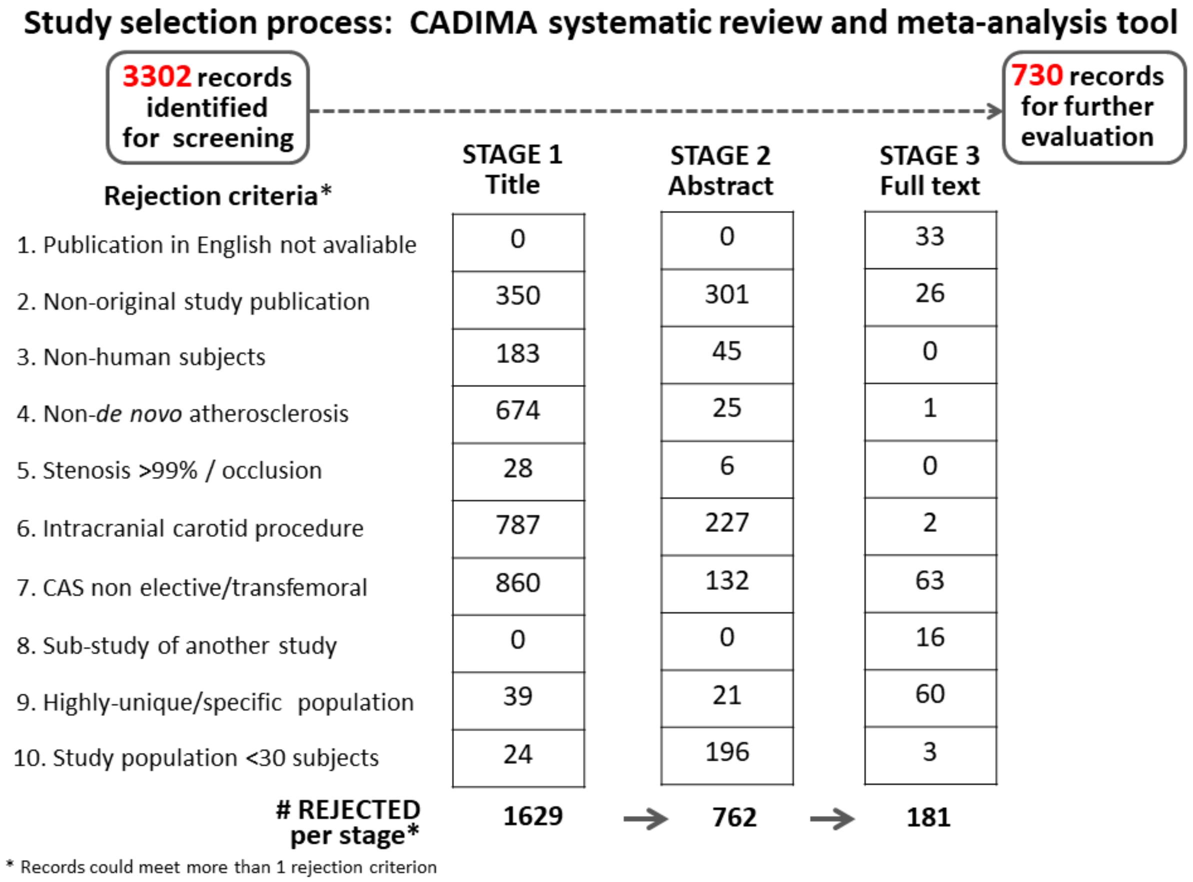

Figure S2. Study selection process: CADIMA systematic review and mata-analysis tool.

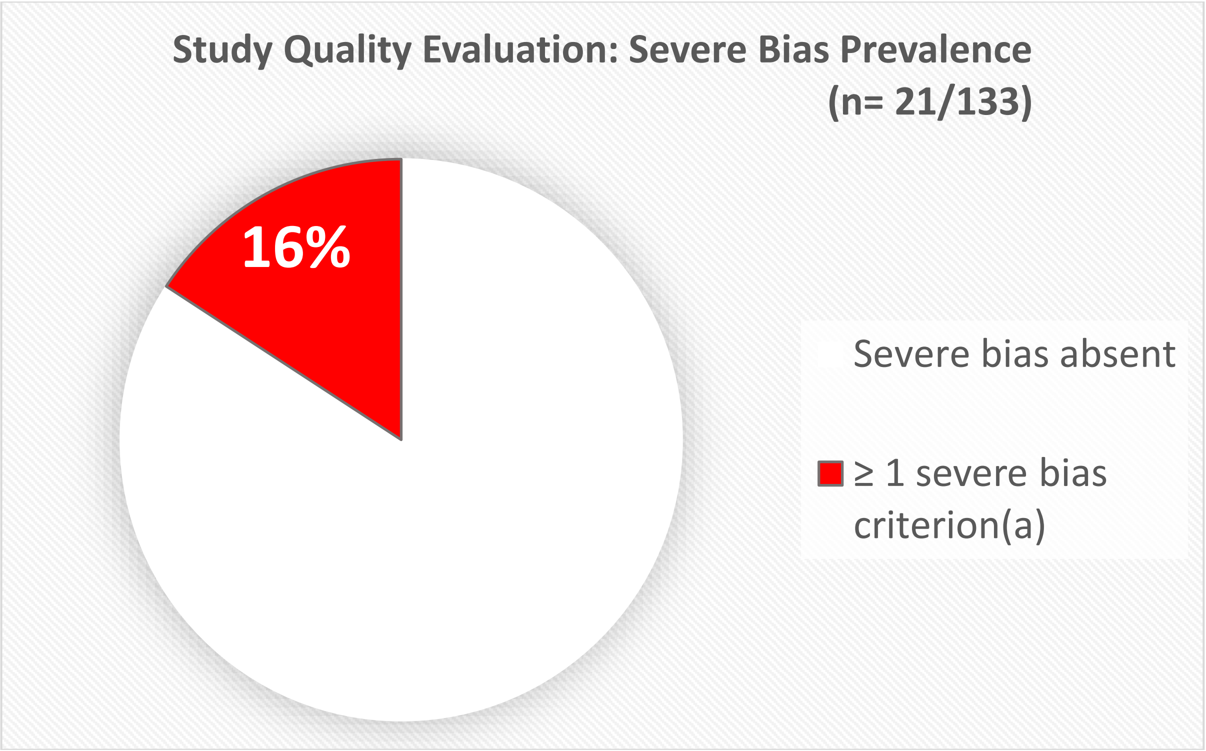
Study Quality Evaluation: Severe Bias Prevalence
(n= 21/133)
16%
Severe bias absent
≥ 1 severe bias criterion(a)

(A)

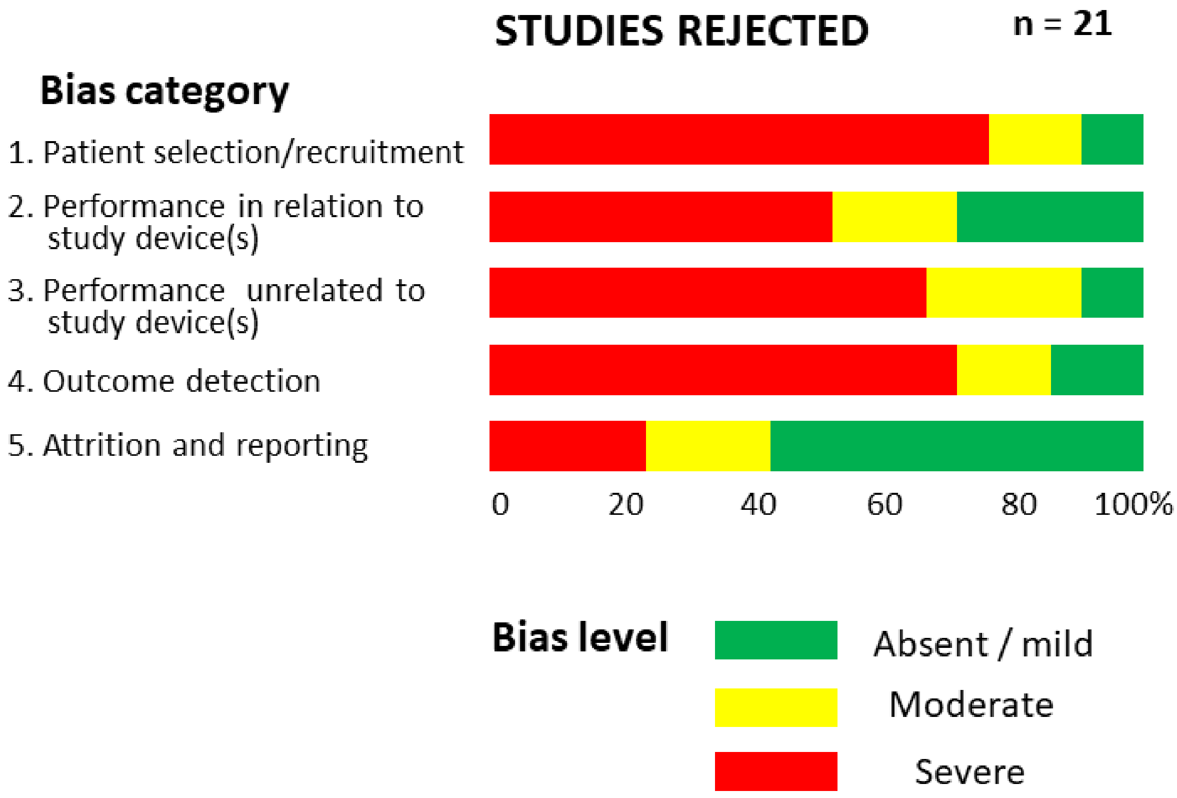
STUDIES REJECTED
n = 21
Bias category
1. Patient selection/recruitment
2. Performance in relation to study device(s)
3. Performance unrelated to study device(s)
4. Outcome detection
5. Attrition and reporting
0
20
40
60
80
100%
Bias level
Absent / mild
Moderate
Severe

(B)

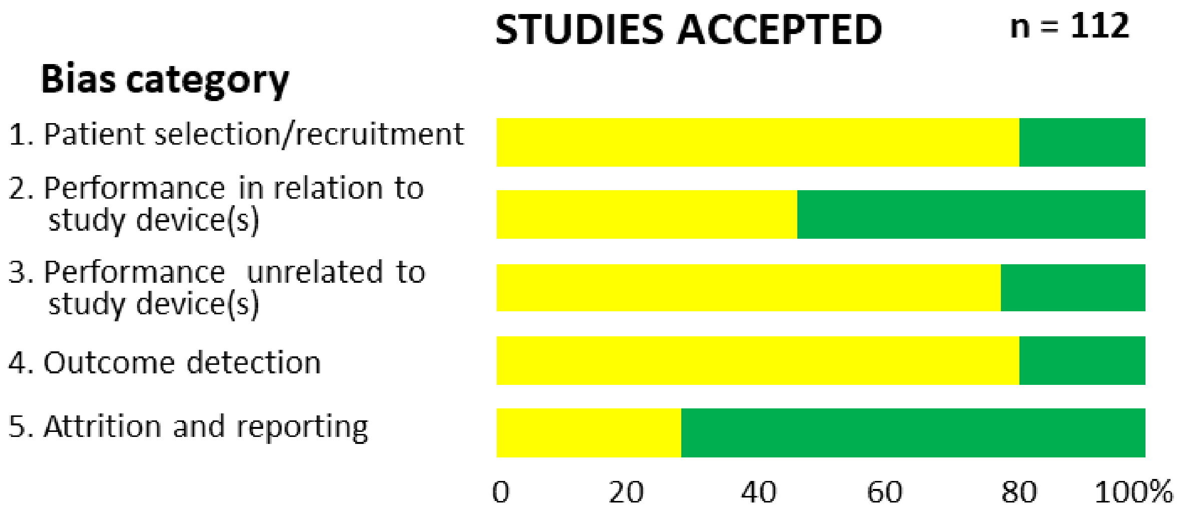
STUDIES ACCEPTED
n = 112
Bias category
1. Patient selection/recruitment
2. Performance in relation to study device(s)
3. Performance unrelated to study device(s)
4. Outcome detection
5. Attrition and reporting
0
20
40
60
80
100%

(C)

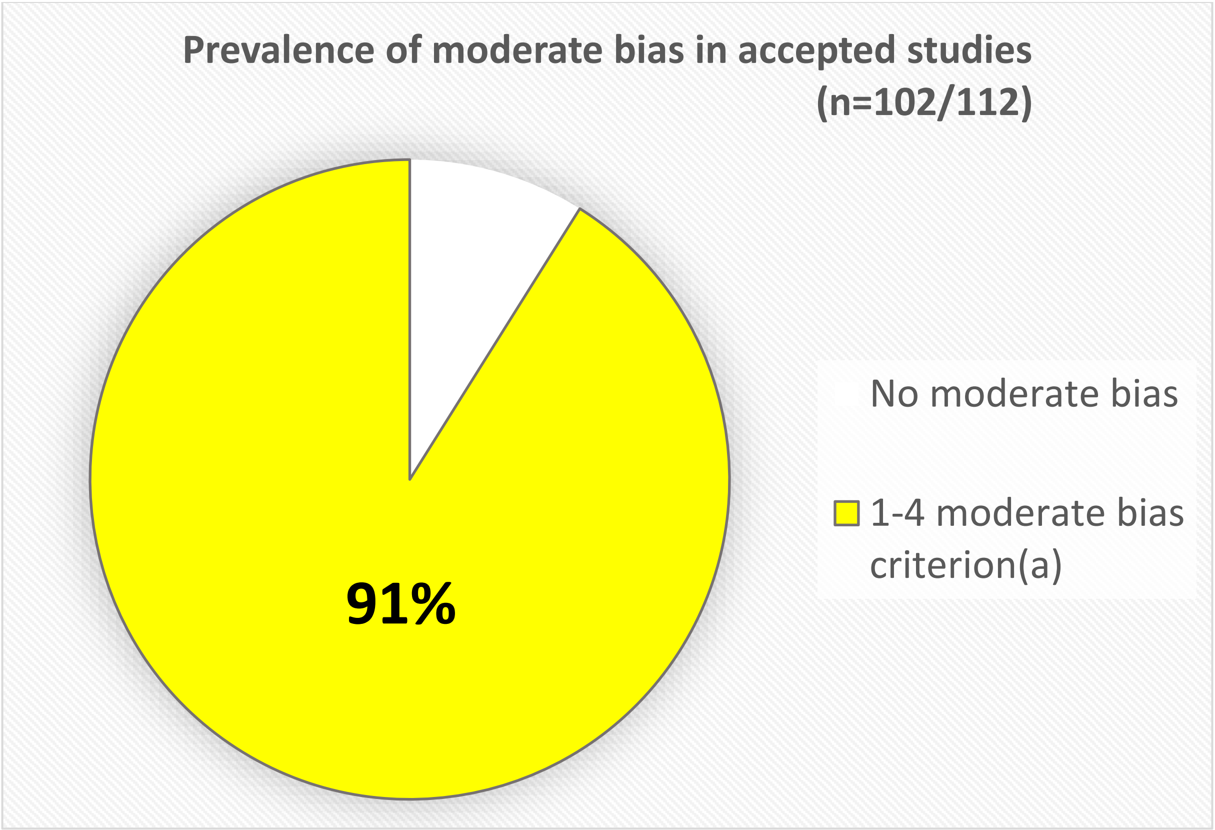
Prevalence of moderate bias in accepted studies (n=102/112)
91%
No moderate bias
1-4 moderate bias criterion(a)

(D)

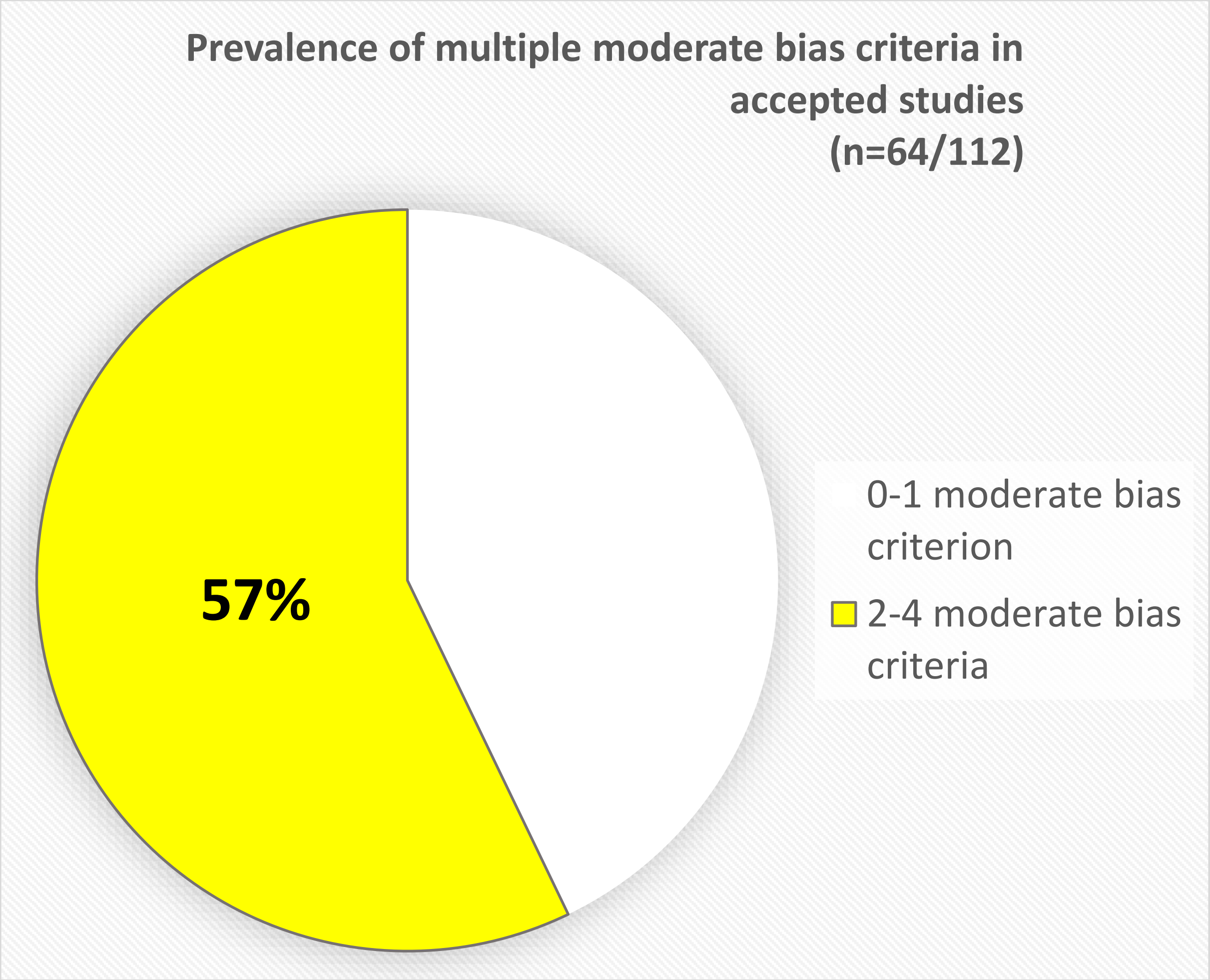

(E)

Figure S3. Bias systematic assessment.

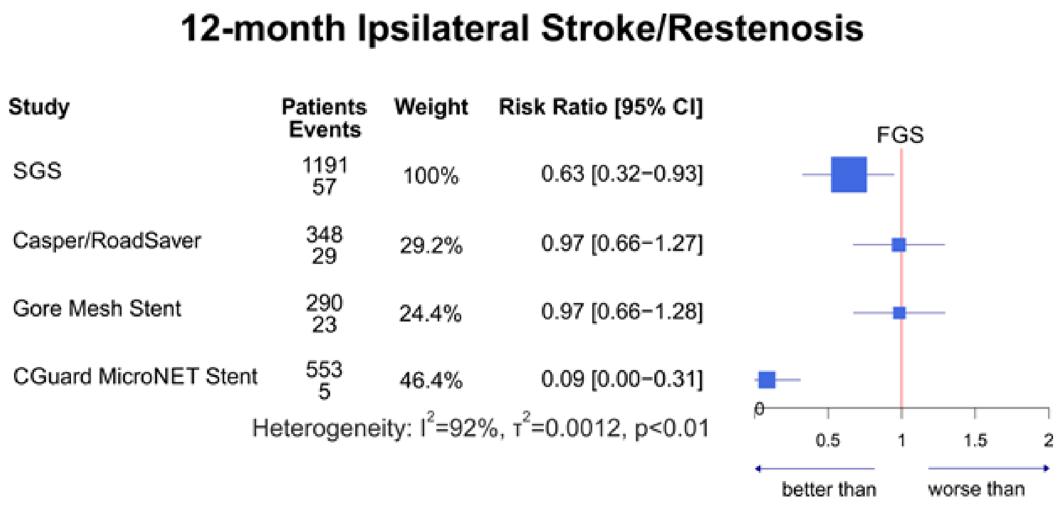

Figure S4. Forrest-plot presenting meta-analytic data for the combined 12-month endpoint of ipsilateral stroke and restenosis.

With FGS used as a reference, the benefit of Casper/Roadsaver in reducing 12-month ipsilateral stroke rate was neutralized by its relative harm—increased restenosis rate. For separate presentation of the individual (rather than combined) endpoints see Figure 2.

30-day Death/Stroke/MI Funnel Plots

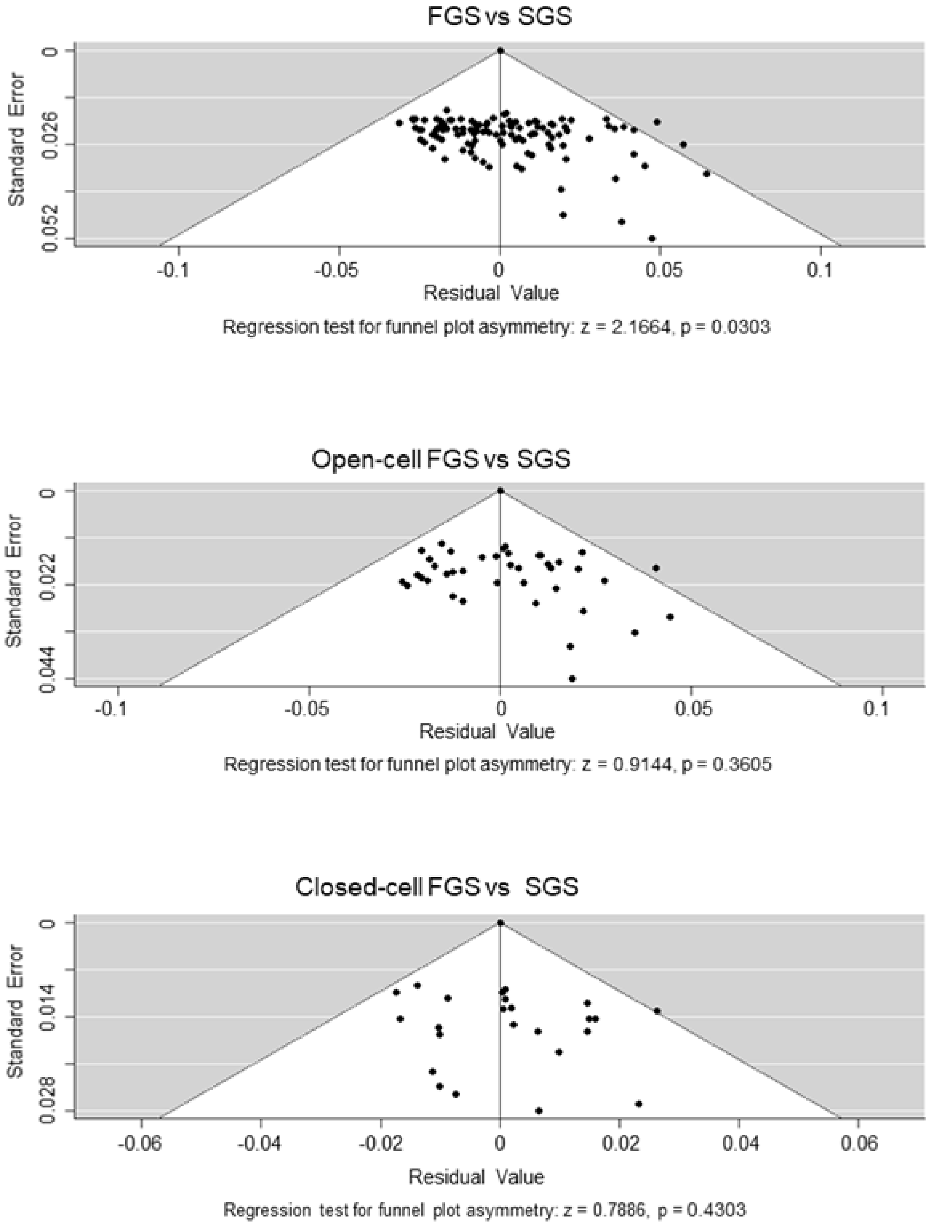

(A)

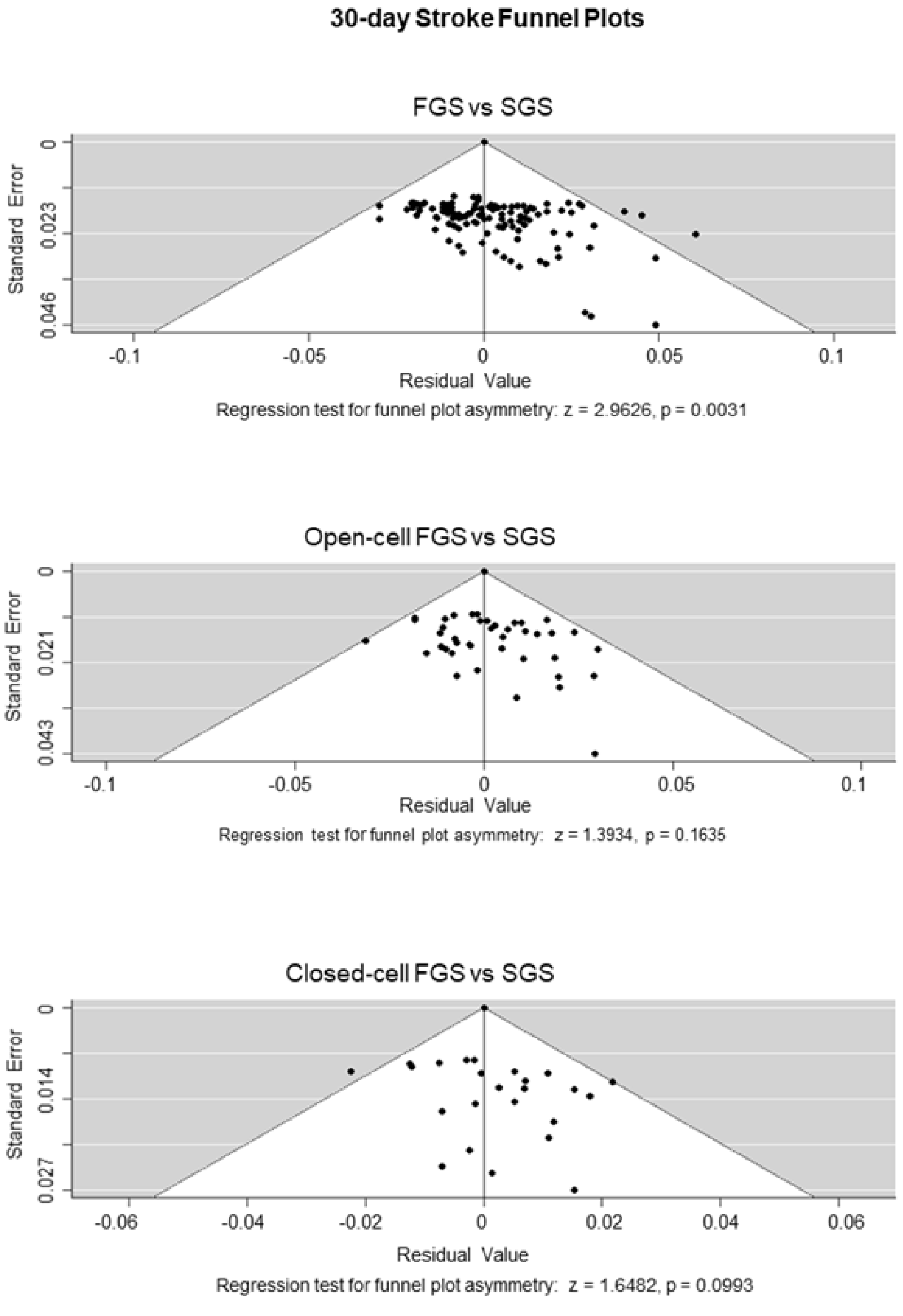

(B)

Figure S5. Funnel plots of different stent type comparisons - 30-day-outcomes. (A) 30-day composite endpoint for death/stroke/MI, (B) 30-day stroke.

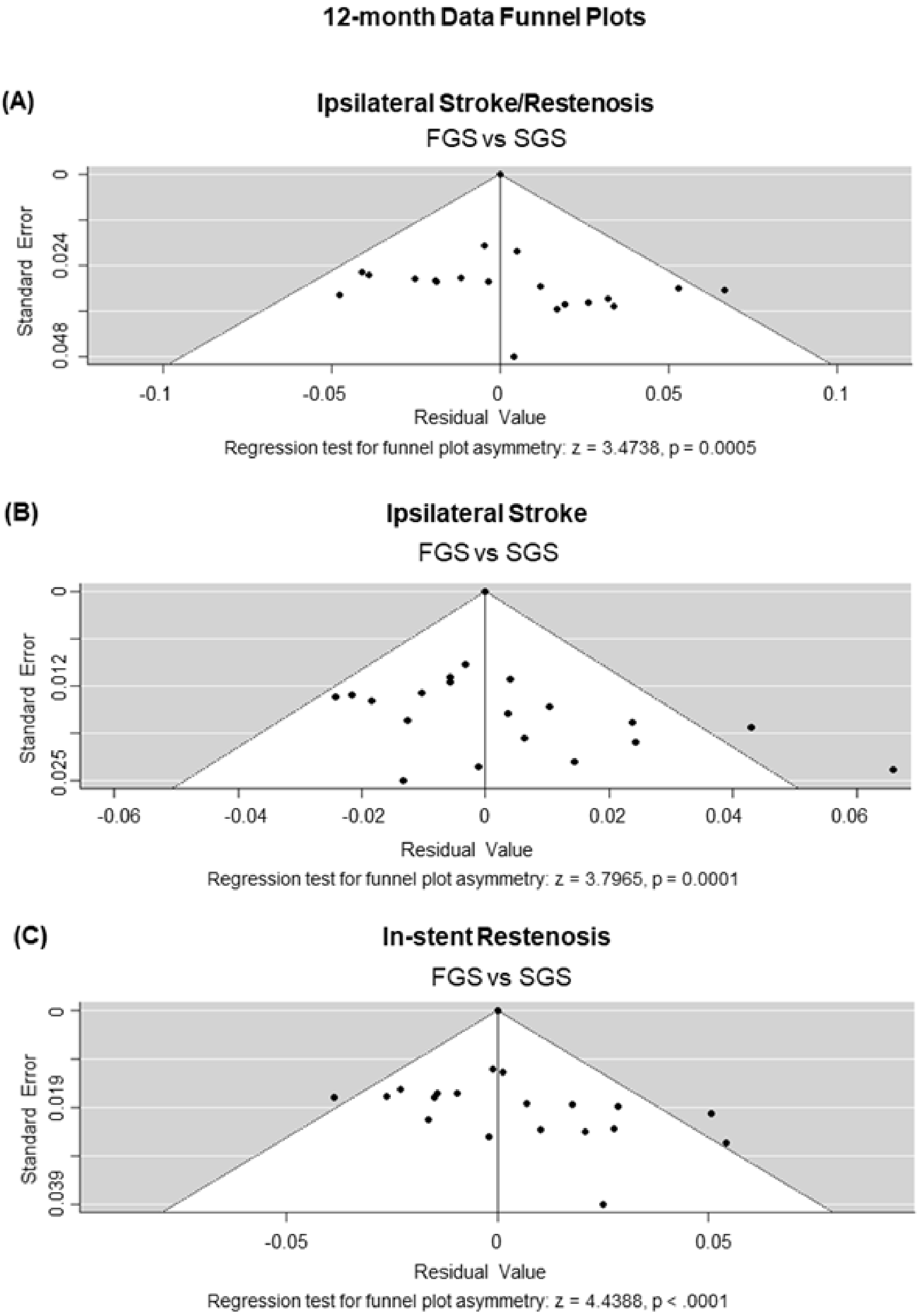

Figure S6. Funnel plots of different stent type comparisons - 12-month outcomes.

CARMEN Investigators are Max Amor, Laura Capoccia, Fausto Castriota, Alberto Cremonesi, Gianmarco de Donato, Tomasz Drazkiewicz, Iris Q Grunwald, Andrey Karpenko, Ralph Kolvenbach, Waclaw Kuczmik, Krzysztof Piotr Malinowski, Adam Mazurek, David Christopher Metzger, Antonio Micari, Andre Monteiro, Piotr Musialek, Dimitrios Nikas, Piotr Paluszek, Ivo Petrov, Piotr Pieniążek, Anna Podlasek, Petar Polomski, Kenneth Rosenfield, Andrej Schmidt, Joachim Schofer, Carlo Setacci, Francesco Setacci, Adnan H. Siddiqui, Horst Sievert, Pasqualino Sirignano, Francesco Speziale, Eugenio Stabile, Lukasz Tekieli, Mariusz Trystuła, Christian Wissgott.
